# Multiple Plant Regeneration from Embryogenic Calli of *Paulownia tomentosa* (Thunb.) Steud

**DOI:** 10.3390/plants11081020

**Published:** 2022-04-08

**Authors:** Aigul Amirova, Symbat Dossymbetova, Yeldana Rysbayeva, Bakdaulet Usenbekov, Arman Tolegen, Alibek Ydyrys

**Affiliations:** 1 Faculty of Biology and Biotechnology, Al-Farabi Kazakh National University, al-Farabi Av. 71, Almaty 050040, Kazakhstan; bakdaulet7@yandex.ru (B.U.); tolegenarman7@gmail.com (A.T.); ydyrys.alibek@gmail.com (A.Y.); 2 Department of Food Technology, Almaty Technological University, Tole be 100, Almaty 050008, Kazakhstan; symbat_89@list.ru (S.D.); eldana-90@mail.ru (Y.R.); 3 Biomedical Research Centre, Al-Farabi Kazakh National University, al-Farabi Av. 71, Almaty 050040, Kazakhstan

**Keywords:** *Paulownia tomentosa*, plant growth regulators, *in vitro*, somatic embryogenesis, plant regeneration

## Abstract

The aim of this paper was to study the effect of plant growth regulators on callus induction and *in vitro* morphogenesis using various explants of *Paulownia tomentosa* to develop an efficient plant regeneration protocol. Different plant organ sections (leaves, apical shoot tips, petals, nodes, and internodes) were cultured as explants to identify the best *in vitro* explants responsive to callus induction and plant regeneration. Explants were cultivated on MS media supplemented with different concentrations of plant growth regulators (TDZ (Thidiazuron), BAP (6-Benzylaminopurine), kinetin, and NAA (1-Naphthaleneacetic acid). It was discovered that the addition of TDZ and NAA stimulated the induction of somatic embryogenesis. It was discovered that the MS medium with the combination of plant growth regulators BAP (35.5 µM) and NAA (5.4 µM) with the addition of 30.0 g/L maltose, 500.0 mg/L casein hydrolysate, and 250.0 mg/L L-proline was optimal for callus induction and multiple plant regeneration. The study of the regenerative capacity of various explants of *Paulownia tomentosa in vitro* showed that plant regeneration depends on the type of explant, and occurs in both ways, indirectly, through the formation of callus tissues and directly on the explant, without callus formation. As a result of this study, the efficient reproducible protocol of embryogenic callus formation and multiple shoot induction *in vitro* of *Paulownia tomentosa* was developed. This system provides a clear increase in the frequency of plant regeneration from 36.3 ± 3.4% to 38.6 ± 2.3% per embryogenic callus from leaves and apical shoot tips, respectively.

## 1. Introduction

*Paulownia tomentosa* (Thunb.) Steud is a valuable woody plant belonging to the family Paulowniaceae. It is native to central and western China, and grows in gardens and parks in North America, Japan, and Korea. *Paulownia tomentosa* is a 10–25 m tall tree with a wide crown and large (up to 30 cm long and 25 cm wide) heart-shaped leaves with long petioles. The flowers are pale purple in erect pyramidal inflorescences; flowering occurs before the leaves [1]. *Paulownia* is a unique plant; all parts of its organs are used for different purposes: flowers are a source of medicinal honey, leaves—rich in protein up to 23%, a trunk—a source of finished products (veneer, furniture board, parquet and etc.). It is not only a decorative tree; it is also a multi-purpose plant and a source of renewable energy, due to the high content of cellulose [2,3].

Nowadays, almost all of the territory of the Republic of Kazakhstan is considered a high-risk zone, due to the development of the desertification process caused by anthropogenic and environmental factors. Desertification is a silent and invisible crisis that causes economic, social, and environmental problems. One of the effective measures to prevent desertification, land degradation, and to strengthen soil structure is to increase the planting of trees and forests. The use of biotechnological methods of microclonal propagation and plant regeneration *in vitro* increased plant production. Currently, fast growing trees *Paulownia tomentosa* are widely used to protect the environment, reduce soil erosion, and improve soil fertility [1,4,5]. Many wild plants have sufficient reserves of raw materials in nature, but the rapid reduction of forest areas due to anthropogenic pressure and unplanned development and the excessive use of cultivated plants contribute not only to a decrease in their number, but also to the extinction of a number of species in nature [6], as well as to a decrease in the number of species in biodiversity [7]. Therefore, an alternative way to obtain more raw materials is to grow medicinal plants in botanic gardens or agricultural fields [8,9], as well as using the methods of *in vitro* plant tissue culture. Currently, many scientists are engaged in the cultivation of medicinal plants in various ways [10,11,12], for example, growing and storing them in the system of organic farming without the use of fertilizers and agricultural drugs. Alternatively, growing medicinal plants using new biogums is used in agriculture [13].

Traditionally, *Paulownia* propagates by seed or root cuttings [14,15]. The main disadvantages of vegetative propagation methods are the genetic diversity of the resulting planting material and the duration of the juvenile period. Seeds germinate slowly or quickly lose germination. Propagation by root cuttings is delayed due to the damage and disease caused by pathogenic microorganisms and pests [16].

The use of modern biotechnological methods of *in vitro* plant propagation can overcome these disadvantages and increase the production of plants much better than the traditional method of propagation by seeds and cuttings. Plant cell and tissue culture techniques, such as microclonal propagation, organogenesis, and somatic embryogenesis are mainly used in plant propagation *Paulownia* ‘Shan Tong’ (*P. fortunei* ∗ *P. tomentosa*) *in vitro* [17]. Organogenesis and somatic embryogenesis are two major morphogenetic pathways in tissue culture *in vitro*. Organogenesis is the formation of organs (shoots and roots) in plant tissue culture. Somatic embryogenesis is a process in which the plant somatic cells, under the influence of external factors, suddenly begin to behave like a zygote and pass through various stages of embryogenesis with the formation of bipolar structures that resemble zygotic embryos [18,19,20]. Somatic embryogenesis is the process of the formation of many somatic embryos of single or multicellular origin and is used for the mass production of plants.

One of the ways to develop reproducible plant cell and tissue technologies is to study the cytophysiological features of the regulation of morphogenesis *in vitro* by changing the composition of nutrient media [21]. The use of biotechnological methods of *in vitro* plant propagation significantly expands the possibility of mass production of somatic embryos and regenerated plants. Most of the developed protocols of plant regeneration of *Paulownia* are mainly associated with the use of nodal explants. Thus, the study of regenerative capacity of various types of explants of *Paulownia* (segments of leaves, petioles, internodes, and nodes) showed that the nodal explants have a high regenerative capacity compared to other types of explants [22,23].

Mostly, the nutrient medium of Murashige and Skoog [24] is used for the propagation of different genotypes of *Paulownia in vitro* [17,25,26]. Furthermore, studying the effects of various plant growth regulators (TDZ, BAP, IAA) on the frequency of plant regeneration, different authors obtained data that are slightly different from each other. Some authors reported that the TDZ was more effective when compared to BAP for the regenerated plant induction of *Paulownia* ‘Shan Tong’ (*P. fortunei* ∗ *P. tomentosa*) [17,27,28]. It was observed that the optimal ratio of auxin to cytokinin should be 1:10, which will contribute to the regeneration of shoots. In this ratio, the highest frequency of adventitious shoots formation was discovered [17].

Ipekci (2001) [22] and Litwinczuk and Bochnia (2012) [29] reported that the MS medium supplemented with 4.44 mg/L BAP and 0.54 µM NAA was found to be optimal for the regeneration of multiple shoots from the shoot tip of *Paulownia* in tissue culture *in vitro*. The high efficiency of BAP over the other cytokinin for the formation of multiple shoots of *Paulownia* was reported by Taha (2008) [30].

Another author showed that the addition of 13.9 µM kinetin and 2.68 µM NAA in the culture medium was most effective for the regeneration of shoots of *Paulownia tomentosa* among the various plant growth regulators (BAP, kinetin, zeatin, and NAA). The leaf segments produced the largest number of shoots per explant (12 ± 0.4) in this medium when two types of explants (apical shoot and leaf segments) were used. The addition of 10% of coconut water (CW) to the abovementioned medium increased the number of shoots (up to 18) per culture. In addition, they showed that the rooting of shoots occurred well on half-strengthened MS medium (½ MS) supplemented with 10.7 µM NAA within 12 to 15 days [26].

In this regard, the aim of this paper is to study the effect of various phytohormones on callus induction and plant regeneration processes *in vitro*, to develop an effective reproducible protocol of multiple plant regeneration of *Paulownia tomentosa*.

## 2. Results

### 2.1. Testing of the Various Nutrient Media for Callus Induction and Plant Regeneration

Testing of the various nutrient media for callus induction and plant regeneration capacities of *Paulownia tomentosa* used MS1-MS6 nutrient media supplemented with 7.0 g/L agar, 30.0 g/L maltose, 1000.0 mg/L casein hydrolysate, and 250.0 mg/L L-proline. It is known that the number of apical shoot tips and nodes is limited on plants and it was decided that their use on all tested media is not profitable. *Paulownia* has a lot of large leaves and there were no restrictions in plant material for the isolation of explants. Therefore, only leaf fragments were selected as explants to study the induction of calli on various media (Table 1) and to select the optimal nutrient medium for obtaining the tissue culture of *Paulownia tomentosa*.

Studying the influence of different plant growth regulators on callus induction revealed that the MS2 medium is optimal (95.5 ± 3.9%) among the used nutrient media (Table 1).

### 2.2. Study the Callusogenesis and Morphogenesis of Different Types of Explants

**Figure 1:**
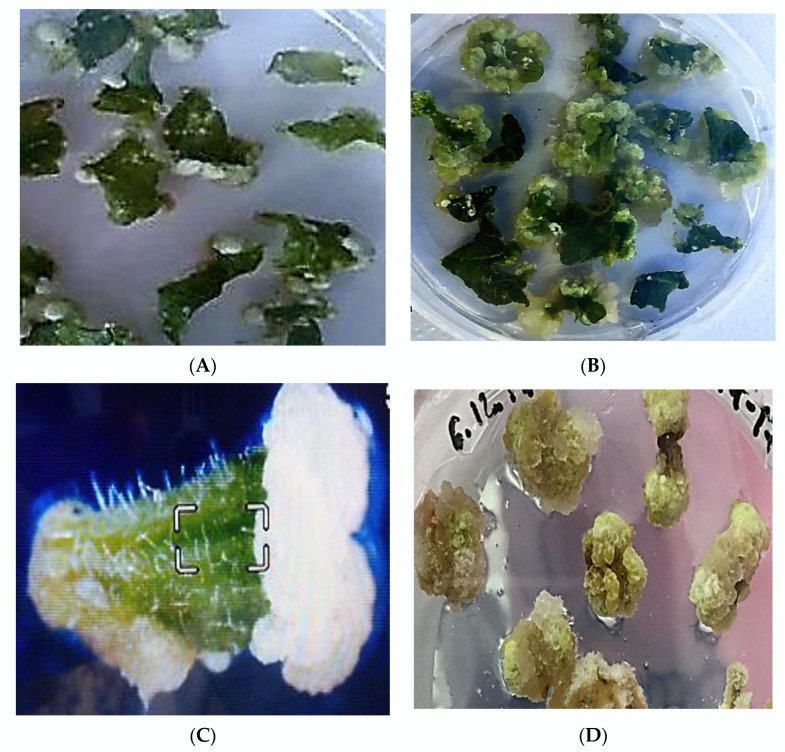

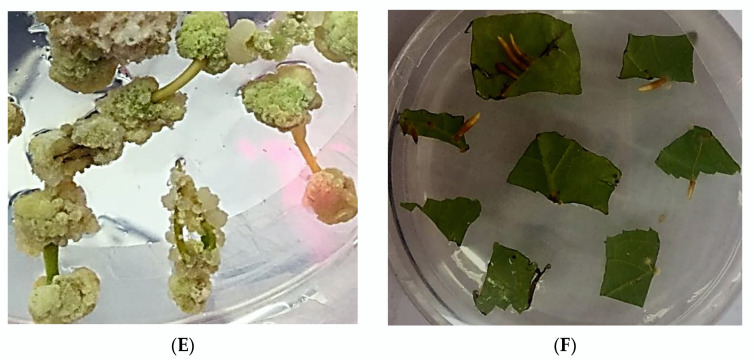
Induction of callus tissues from different explants of *Paulownia tomentosa* on MS2 media: morphology of white callus tissues from leaves (**A,B**), internodes (**C**), nodes (**D**), and petals (**E**); root formation directly (organogenesis) from the explants of leaf on MS media with 13.9 µM kinetin + 2.8 µM IAA (**F**).

Therefore, MS2 was determined as the best medium for the callusogenesis of *Paulownia tomentosa* among the tested media, and it was used to study the callus induction capacities of different explants carried out on MS medium supplemented with 35.5 µM BAP and 5.4 µM NAA. It was concluded that the number of days to initiate callus was dependent on the types of explants. Thus, the induction of primary callus tissues on explants of leaves and apical shoot tips was observed after the 7th–9th day; callus induction from leaves (Figure 1A) and entire leaf explant turns into callus tissue (Figure 1B). The induction of primary calli on explants from petioles, nodes and internodes was observed on the 5th–6th day of cultivation on nutrient media (Figure 1C–E). The white dense globular calli (Figure 1A–E) are formed on different types of explants in this media. The callus induction was not detected on the two MS media with the addition of kinetin and NAA (MS5–MS6), but the initiation of roots directly on explants was observed (Figure 1F).

A high percentage of callus tissues formation was observed in all types of explants. The frequency of callus induction from leaves is greater than others (Table 2). It was found that the leaves are the best explants for callus induction.

The morphological study of calli sorts tissues into two types: dense white globular and white–greenish embryogenic tissues. It is observed that the induction of white dense globular calli begins primarily in the area of the cutting of the explants. During cultivation, white dense globular calli were transformed into white–greenish embryogenic tissues (Figure 1B). It was noticed that after the first subculture calli began growing fast and all explants produced callus tissues.

**Figure 2:**
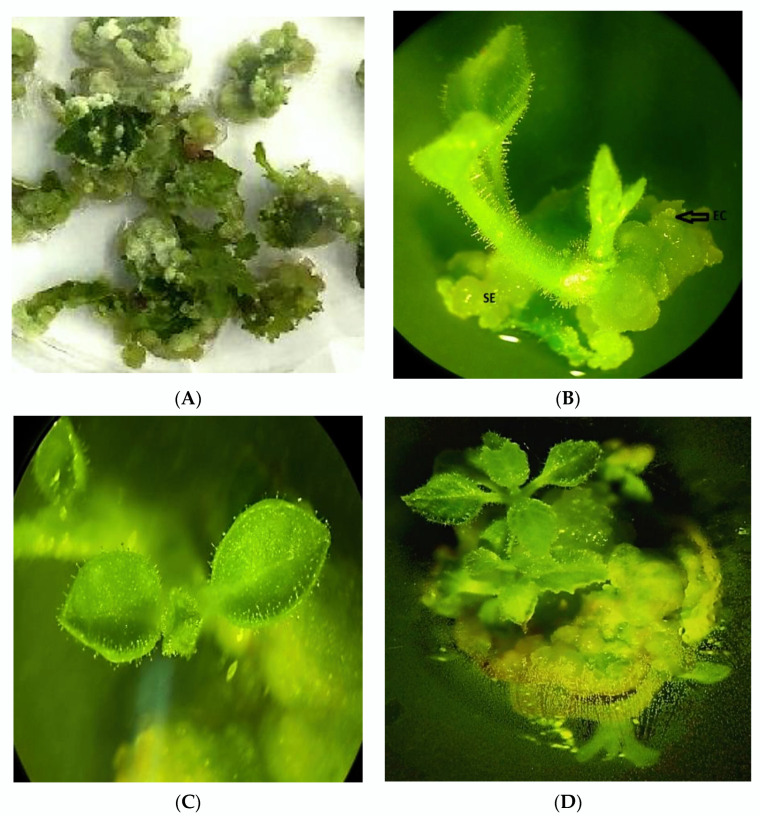
Induction of callus tissues (**A**) and plant regeneration from embryogenic callus from fragments of leaf on MS media with 4.5 µM TDZ + 2.8 µM IAA (**B**–**D**). Symbols in (**B**): SE—somatic embryos, EC—embryogenic calli with small needle-like embryogenic structures at early stages of SE (micro photo).

It was discovered that the most optimal nutrient media for the induction of white–greenish embryogenic calli from leaf explants was MS with a combination of TDZ and NAA. Thus, TDZ stimulates the induction of somatic embryogenesis: the formation of white–greenish embryogenic callus tissues after several subculturing (3–4 passages) and spontaneous regenerated plants (Figure 2A–D) were observed. Figure 2B clearly shows the production of regenerated seedlings from embryogenic calli (EC). Embryogenic calli are characterized by the smallest needle-like embryogenic structures, which further develop and differentiate into somatic embryoids (SE) and give rise to whole plants.

Thus, the addition of TDZ to the nutrient medium stimulates the induction of embryogenic and regenerable callus tissues. It was found that increasing the concentration of TDZ in the medium leads to a decrease in the regenerative potential of calli. Callus induction on the two MS media supplemented with kinetin and NAA was not detected, however, the initiation of roots directly on explants was observed.

### 2.3. Plant Regeneration In Vitro from Different Types of Explants

**Figure 3:**
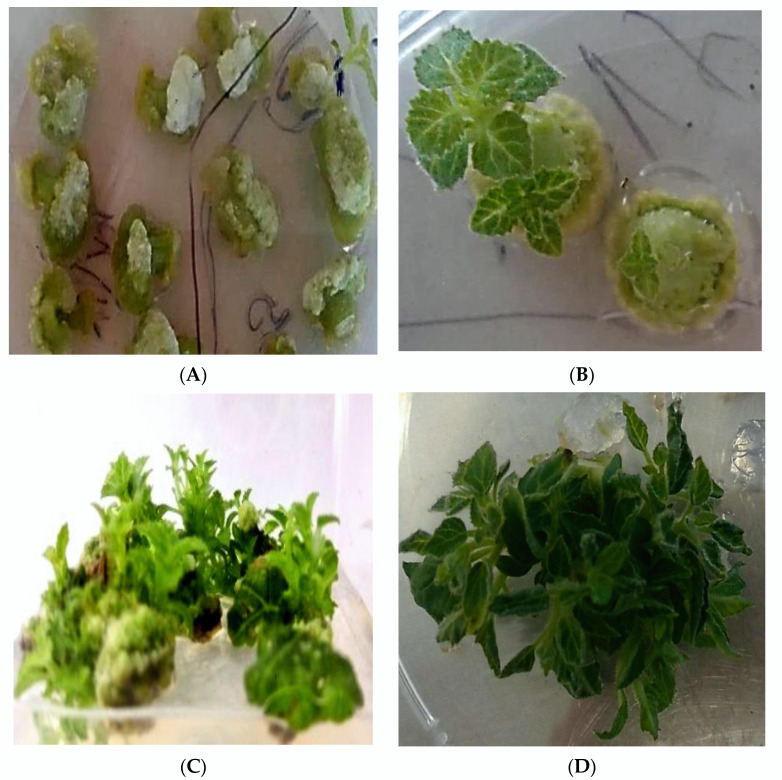
Indirect and direct plant regeneration from different types of explants on MS2 medium: internodes and petals indirectly, through the callus formation (**A**) and directly, without calli formation, on nodal explants (**B**); plant regeneration from leaves (**C**) and apical shoot tips (**D**) through the callus formation.

The study of the regenerative capacity of various explants of *Paulownia tomentosa in vitro* showed that plant regeneration depends on the type of explant and occurs in both ways—indirectly, through the formation of callus tissues, which regenerate plants during the process of the differentiation of embryogenic structures, and directly on the explant, without callus formation. It was established that the leaves and petals firstly formed callus tissues and then regenerated plants were induced from them (Figure 1A–C and Figure 3A,C). Plant regeneration on nodal explants occurs directly, without callus induction. In the case of using the apical shoot tips and internodal explants, plant regeneration occurs in two ways: directly and indirectly (Figure 3B).

During the second and further subcultures (up to 2 years) the multiple plant regeneration from callus tissues occurs on the different explants of *Paulownia tomentosa*.

### 2.4. Selection of Highly Regenerable Embryogenic Calli (EC)

Due to the high-frequency propagation of embryogenic calli and an increasing volume of callus subcultures, we need to select EC lines with high embryogenic and regenerative capacity to increase the efficiency of plant production (Table 3).

In accordance with Table 3, the EC lines with high embryogenic and regenerable capacities from leaf and apical shoot explants were selected. It was revealed that the frequency of regenerated plants of *Paulownia tomentosa* stretched from 7.4 ± 2.7 in II passage to 36.3 ± 3.4 plants per callus in VI passage of EC lines from leaf explant (Table 3). The number of regenerated plants from EC lines originated from apical shoot tips achieved from 9.1 ± 2.1 to 38.6 ± 2.3 in VI passage. The EC lines kept their ability to regenerate plants for a long period of time (2 years). It should be noted that, in order to increase the yield of plants from apexes (their number is limited in plants), the apical part of the shoots was divided into 2–3 parts with a sterile blade or scalpel.

Therefore, the highest regeneration capacity of callus tissues was observed on MC2 medium containing 35.5 µM BAP and 5.4 µM NAA. This medium was selected as optimal for plant regeneration from the tested nutrient media. It was concluded that the leaves and apical shoot tips are the best explants for somatic embryogenesis and plant regeneration. Of course, we also successfully used other types of explants. The number of regenerated plants increased with the time of subcultures of calli and plant regeneration capacity, continuing for up to 2 years.

### 2.5. Rooting and Acclimatization of Regenerated Plants

**Figure 4:**
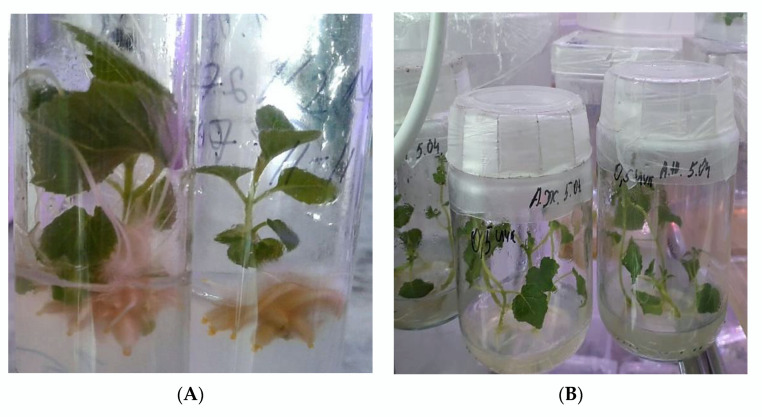

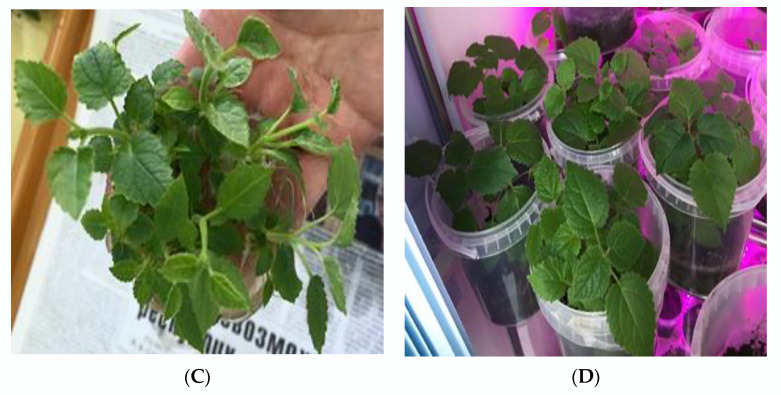
Rooting of regenerated plants on ½ MS media with 5.4 µM NAA and transfer of rooted plants to soil: well rooted shoots *in vitro* (**A,B**), after rooting plants transferred to the soil (**C,D**).

Regenerated shoots were rooted well (100%) in all rooting media, but these media were distinguished by the time of root initiation. The root induction was observed after 20–30 days in the presence of low concentrations of NAA (2.7 µM) or IAA (2.8 µM) in the media without plant growth regulators in the rooting medium. The rooting of regenerated plants (Figure 4A,B) started after 6–8 days of culturing on media ½MS with 5.4 µM NAA or 5.7 µM IAA.

Increasing the concentration of IAA and NAA up to 11.4 µM and 10.7 µM, respectively, prolonged the rooting of shoots. The appearance of roots was observed after 10–15 days in media with a high concentration of auxins. It was determined that a high concentration of auxins promotes the rapid rooting of shoots. The successful and rapid rooting of shoots was demonstrated on media ½MS with 5.4 µM NAA or 5.7 µM IAA. Thus, the media ½MS with 5.4 µM NAA or 5.7 µM IAA accelerated the rooting of regenerated plants of *Paulownia tomentosa* (Figure 4A–D).

An average (90%) of adaptation of the regenerated plants was achieved when they were transferred from *in vitro* to glasses with water. The well acclimatized plants were transferred to pots with soil, peat, and perlite in a ratio of 1:1:1 (Figure 4D). However, the transportation of acclimatized plants to the soil was the most critical and difficult stage, at which not all plant regenerants survived. We achieved an average survival rate (98%) in vivo of regenerated plants. Then, plants were transferred to the greenhouse.

## 3. Discussion

The use of biotechnological propagation methods allows one to increase the yield of plants in comparison with the traditional method of seed propagation. In this article, experiments are devoted to the development of methods to increase the yield of the regeneration of plants of *Paulownia tomentosa* in tissue culture *in vitro*.

In the available literature, it is known that the well-known nutrient medium Murashige and Skoog [24] is used for the *in vitro* tissue culture and plant regeneration of *Paulownia tomentosa*, and among the plant growth regulators, TDZ, BAP, and IAA are used.

As a result of our experiments, it was discovered that the addition of TDZ to the nutrient medium stimulates the induction of somatic embryogenesis. However, increasing the concentration of TDZ leads to decreasing the regenerative potential of calli. The induction of callus tissues on media supplemented with kinetin and NAA was not shown, but an increase of roots directly on the explants was observed.

Among the tested six media with various plant growth regulators, media with a combination of 35.5 µM BAP and 5.4 µM NAA turned out to be the most optimal for callus induction, somatic embryogenesis, and plant regeneration.

Studying the regenerative capacities of different explants (leaves, apical shoot tips, petioles, nodes, and internodes) showed two pathways of plant regeneration *in vitro*: direct and indirect. Direct plant regeneration on explants is rare; mostly an indirect pathway of plant regeneration through callus formation was found in the *in vitro* tissue culture of *Paulownia tomentosa*. The earlier, direct, and indirect plant regeneration from leaf and internode explants in *Paulownia elongata* was reported by Ipekci and Gozukirmizi [31,32]. Most of the developed protocols of plant regeneration of *Paulownia* are mainly associated with the use of nodal explants. Thus, the study of the regenerative capacity of various types of explants of *Paulownia* (segments of leaves, petioles, internodes, and nodes) indicates that the nodal explants show a high regenerative capacity compared to other types of explants [22,23].

Ipekci [22] and Litwinczuk and Bochnia [29] reported that the MS medium supplemented with 4.4 µM BAP and 0.54 µM NAA was found optimal for the regeneration of multiple shoots from the shoot tip of *Paulownia tomentosa* in tissue culture *in vitro*. A high efficiency of BAP over the other cytokinin for the formation of multiple shoots of *Paulownia* was reported by Taha [30]. Pozoga et al. presented a high potential of *in vitro* micropropagation of *Paulownia tomentosa* ∗ *Paulownia fortunei* hybrid by inducing organogenesis from nodular explants on ½ MS media with various concentrations of BAP [33]. Rahman et al. studied the propagation of the *Paulownia tomentosa* tree *in vitro* from apical shoot and lateral bud explants on MS media containing various plant growth regulators: BAP, NAA, and kinetin. The authors achieved a high percentage of plant induction (84%) on MS medium with 11.1 µM BAP + 2.68 µM NAA, where the plant yield was 7.4 shoots per explant [34]. Roy (2015) [26] showed that the addition of 13.9 µM kinetin and 2.68 µM NAA in the culture medium was effective for the regeneration of shoots of *Paulownia tomentosa* (Thunb.) Steud. among the various plant growth regulators (BAP, kinetin, zeatin and NAA). The leaf segments produced the largest number of shoots per explant (12 ± 0.4) in this medium when two types of explants were used (apical shoot and leaf segments). The addition of 10% of coconut water (CW) to the abovementioned medium increased the number of shoots (up to 18) per culture. In addition, they showed that the rooting of shoots was good on half strengthened MS medium (½ MS) supplemented with 10.7 µM NAA within 12 to 15 days [26]. As can be seen, many researchers of the *in vitro* propagation of *Paulownia* frequently used the MS medium, and based on this, the authors of this paper selected this medium for their experiments. In this experiment, authors used plant growth regulators BAP and NAA, which stimulated the induction of somatic embryogenesis and plant regeneration.

The tissue culture system described here provides a highly efficient and reproducible protocol of the embryogenic callus induction and multiple shoot regeneration of *Paulownia tomentosa in vitro*. The selection of EC lines with high embryogenic and plant regeneration capacities from leaf and apical shoot explants accelerated and increased the production of plants *Paulownia tomentosa in vitro*. Thus, the system developed by us provides a clear increase in the frequency of plant regeneration from 36.3 ± 3.4 to 38.6 ± 2.3 per callus (EC) originating from leaves and apical shoot tips, respectively, and a significantly higher percentage of calli showing the regeneration potential relative to levels reported in the literature on the tissue culture of *Paulownia tomentosa*. These lines of EC tissue retain the ability to regenerate plants for a long period. Moreover, the developed protocol for multiple regenerations of plants from embryogenic calli can be applied to genetic manipulations of *Paulownia tomentosa*. Moreover, there is an attempt to obtain a transgenic woody plant of *Paulownia tomentosa* resistant to bacterial infections [35].

## 4. Materials and Methods

### 4.1. Plant Material

The object of the study is the plants of *Paulownia tomentosa* (Thunb.) Steud. The various parts of plant organs (leaf, apical shoot tip, petal, node and internode) were used as explants for introduction into *in vitro* culture. This experimental work was carried out at the Department of Food Biotechnology of Almaty Technological University in 2018–2019. The process of writing and designing the article began after transferring to the Faculty of Biology and Biotechnology of Al-Farabi Kazakh National University.

### 4.2. In Vitro Culture Techniques

The conventional methods of plant *in vitro* tissues culture [36] were used to obtain the callus tissues and plant regeneration of *Paulownia tomentosa*. MS medium served as a basal medium for all experiments, since in the literature, this medium is often used for obtaining the tissue culture of *Paulownia tomentosa*. Various concentrations of plant growth regulators (auxins and cytokinins) were added to the nutrient medium. Explants were surface-sterilized by exposure to 70% ethanol (1 min), followed by 0.5% sodium hypochlorite (7 min) and rinsing with sterile distilled water (three times).

After sterilization, explants were excised under aseptic conditions into segments 0.7–0.9 cm in size and placed for callus induction on solid nutrient media (Murashige and Skoog (1962)), with the addition of various concentrations of plant growth regulators (TDZ (Sigma-Aldrich, St. Louis, MO, USA), NAA (Sigma-Aldrich, USA), BAP (Sigma-Aldrich, USA), and kinetin (Sigma-Aldrich, USA)). In addition to phytohormones, culture media (MS1–MS6) were supplemented with 30.0 g/L of maltose, 1000.0 mg/L of casein hydrolysate and 250.0 mg/L of L-proline, and solidified with 7.0 g/L agar (suitable for plant tissue culture, Sigma-Aldrich, USA). Maltose (30 g/L) was used as a carbon source. It is known that maltose stimulates the process of morphogenesis in tissue culture *in vitro*. Therefore, 30.0 g/L of maltose was added to the composition of nutrient media instead of sucrose, which is usually used for plant tissue culture media. L-proline and amino acid complexes such as casein hydrolysate were added to all nutrient media. Casein hydrolysate and L-proline are essential for the induction of somatic embryogenesis in tissue culture *in vitro*. In the literature, these substances are added to nutrient media for the stimulation of somatic embryogenesis (SE) in the *in vitro* culture of different plant species [37,38,39]. Vitamins (thiamin (B1), nicotinic acid, and pyridoxine (B6)) and other additives (inositol and glycine) were added to the used nutrient media in accordance with the standard chemical composition of MS medium. After the addition of plant growth regulators, the pH of the media was adjusted to 5.8 with solutions of 1N HCL and 1N NaOH before autoclaving. Then, the nutrient media were sterilized by autoclaving at a pressure of 1 atm, the duration of sterilization—30 min.

Isolated *in vitro* plant tissues were cultured under strictly controlled conditions: under light 50–60 mmol m^−2^ s^−1^. photoperiod (16/8 h) and temperature (24–26 °C). A subculture was carried out on callus induction medium every 2 weeks.

### 4.3. Callus Induction and Plant Regeneration

Callus formations on these explants started after 5–9 days. Spontaneous plant regeneration was observed on callus induction medium MS2 after 2 subcultures. The morphology of callus tissues was studied visually and under a stereomicroscope “Technival 2” (Carl Zeiss Jena). Plant regeneration was observed after a second subculture on the callus induction medium.

### 4.4. Testing of the Various Nutrient Media for Callus Induction and Plant Regeneration

The study was carried out in 2 stages. In the first experiment, different types of nutrient media (MC1–MC6) were tested using one type of explants in order to select the best medium for callus induction and plant regeneration. Only leaf fragments were cultured on various nutrient media (MS1–MS6). The callus induction frequency was determined by the proportion of explants number that formed callus tissues. The frequency of regeneration capacities was represented by the number of regenerated plants on the variant of the experiments.

### 4.5. Study the Callusogenesis and Morphogenesis of Different Types of Explants

In the second experiment, the callus formation and plant regeneration capacities of different types of explants (apical shoots, nodes, internodes) of *Paulownia tomentosa* were studied on the optimal nutrient medium which was selected in the first experiment.

### 4.6. Rooting and Acclimatization of Regenerated Plants

The rooting of regenerated plants was supported on different media containing half-strength mineral salts of MS medium and 20.0 mg/L sucrose, and with the addition of various concentrations of NAA (2.7, 5.4, 10.7 µM) or IAA (2.8, 5.7, 11.4 µM) and hormone-free ½ MS medium. Then, regenerated plants 3–5 cm in height and a well-developed root system after washing from agar were transferred to glasses with water (water was changed every day). Regenerated plants were covered with polyethylene lids, and sometimes the cover in order to gradually acclimatize the plants. Well rooted plantlets were transferred to pots with soil, peat, and perlite in a ratio of 1:1:1 and cultivated in the greenhouse under a 16/8 h (day/night) photoperiod, and at a temperature of 24 ± 2 °C.

### 4.7. Statistical Analysis

In each experiment for the testing of different nutrient media or the studying of callus formation and plant regeneration capacities of different types of explants, 10–12 explants per one Petri dish were used. Then, the obtained calli were separated from the explants, divided into several parts, and 12–14 callus fragments were placed on a fresh nutrient medium for multiplication. In each variant of the experiment, three Petri dishes were used, and 3–4 replicates were repeated.

The callus induction and plant regeneration rates were represented as the proportions of explants number that produced callus tissues or plant regeneration (mean value ± standard deviation). The statistical analysis of experimental data was carried out by determining the mean value and standard deviation and expressed as a percentage: the number of calli ∗ 100%/the number of explants.

## 5. Conclusions

In conclusion, an efficient and reproducible protocol of the *in vitro* propagation of *Paulownia tomentosa* has been developed. High embryogenic callus formation and multiple shoot regenerations *in vitro* of *Paulownia tomentosa* were observed. We recommend this reproducible technology of somatic embryogenesis and plant regeneration to use in the propagation of valuable woody plants, to study the molecular and cellular mechanisms of somatic embryogenesis and plant regeneration *in vitro*, and to use it in the genetic transformation of *Paulownia tomentosa.*

## Figures and Tables

**Table 1:** The influence of media composition on callus induction and plant regeneration from leaves of *Paulownia tomentosa*.

Culture Medium	Concentration of Plant Growth Regulators	Callus Induction, %	Plant Regeneration, %
MS1	22.2 µM BAP + 5.4 µM NAA	87.6 ± 2.6	44.4 ± 0.8
MS2	35.5 µM BAP + 5.4 µM NAA	95.5 ± 3.9	95.2 ± 1.9
MS3	4.5 µM TDZ + 2.8 µM IAA	82.4 ± 4.3	23.9 ± 2.5
MS4	13.6 µM TDZ + 2.8 µM IAA	80.2 ± 3.7	12.5 ± 1.8
MS5	13.9 µM kinetin + 2.8 µM IAA	0	0
MS6	23.2 µM kinetin + 2.8 µM IAA	0	0

**Table 2:** The callus induction and plant regeneration capacities of calli from different types of explants of *Paulownia tomentosa* on MS2 medium.

Explants Type	Induction of Callus Tissues, %	Plant Regeneration, %
Leaves	95.5 ± 3.9	95.2 ± 1.9
Apical shoot tip	83.3 ± 14.5	87.2 ± 2.9
Nodes	78.6 ± 6.1	45.3 ± 2.0
Internodes	87.8 ± 9.0	37.6 ± 0.8
Petals	73.9 ± 7.2	34.7 ± 1.5

**Table 3:** Relationship between the number of passages and plant regeneration in embryogenic calli lines of *Paulownia tomentosa*.

Culture Media	Embryogenic Callus (EC) Types	Shoot Number Per 1 EC Number of Passages			
		II	IV	VI	X
MS2	EC (from leaves)	7.4 ± 2.7	27.3 ± 2.2	36.3 ± 3.4	22.8 ± 1.9
	EC (apical shoot tips)	9.1 ± 2.1	29.6 ± 4.2	38.6 ± 2.3	23.6 ± 2.0

## Data Availability

All the data are included in the present study.

## References

[1] Erbar C., Gülden C. (2011). Ontogeny of the flowers in *Paulownia tomentosa*—A contribution to the recognition of the resurrected monogeneric family Paulowniaceae. Flora Morphol. Distrib. Funct. Ecol. Plants.

[2] Yadav N.K., Vaidya B.N., Henderson K., Lee J.F., Stewart W.M., Dhekney S.A., Joshee N. (2013). A Review of *Paulownia* Biotechnology: A Short Rotation, Fast Growing Multipurpose Bioenergy Tree. Am. J. Plant Sci..

[3] Barton I.L., Nicholas I.D., Ecroyd C.E. (2007). Paulownia. For. Res. Bull.

[4] Stupin D.Y. (2009). Soil Pollution and the Latest Technologies for Their Restoration: Training Manual.

[5] Merkle S.A. (2006). Engineering Forest Trees with Heavy Metal Resistance Genes. Silvae Genet..

[6] Ydyrys A., Abdolla N., Seilkhan A.S., Masimzhan M., Karasholakova L. (2020). Importance of the geobotanical studying in agriculture (with the example of the Sugaty region). E3S Web Conf..

[7] Ydyrys A., Yeszhanov B., Baymurzaev N., Sharakhmetov S., Mautenbaev A., Tynybekov B., Baidaulet T. (2020). Technology of landscaping in arid zones by using biohumus from sheep wool. E3S Web Conf..

[8] Khan U.M., Sameen A., Aadil R.M., Shahid M., Sezen S., Zarrabi A., Ozdemir B., Sevindik M., Kaplan D.N., Anitha T. (2021). Citrus Genus and Its Waste Utilization: A Review on Health-Promoting Activities and Industrial Application. Evid.-Based Complementary Altern. Med..

[9] Painuli S., Quispe C., Herrera-Bravo J., Semwal P., Martorell M., Almarhoon Z.M., Rad J.S., Alshehri M.M., Daştan S.D., Taheri Y. (2022). Nutraceutical Profiling, Bioactive Composition, and Biological Applications of *Lepidium sativum* L. Oxidative Med. Cell. Longev..

[10] Taheri Y., Quispe C., Herrera-Bravo J., Sharifi-Rad J., Ezzat S.M., Merghany R.M., Shaheen S., Azmi L., Mishra A.P., Sener B. (2022). *Urtica dioica*-Derived Phytochemicals for Pharmacological and Therapeutic Applications. Evid.-Based Complementary Altern. Med..

[11] Ydyrys Y., Serbayeva A., Dossymbetova S., Akhmetova A., Zhuystay A. (2020). The effect of anthropogenic factors on rare, endemic plant species in the Ile Alatau. E3S Web Conf..

[12] Ydyrys A., Zhaparkulova N., Aralbaeva A., Mamataeva A., Seilkhan A., Syraiyl S., Murzakhmetova M. (2021). Systematic Analysis of Combined Antioxidant and Membrane-Stabilizing Properties of Several *Lamiaceae* Family Kazakhstani Plants for Potential Production of Tea Beverages. Plants.

[13] Bukenova E.A., Bassygarayev Z.M., Akhmetova A.B., Zhunusbayeva Z.K., Ydyrys A. (2019). Development of the method of obtaining the endogenic biostimulator from wheat green spike glumes. Res. Crops.

[14] Rao C., Goh C., Kumar P.P. (1996). High frequency adventitious shoot regeneration from excised leaves of *Paulownia spp*. cultured *in vitro*. Plant Cell Rep..

[15] Ozaslan M., Can C., Aytekin T. (2005). Effect of explant source on *in vitro* propagation of *Paulownia tomentosa* Steud. Biotechnol. Biotech. Equipment..

[16] Bergmann B.A., Moon H.K. (1997). *In vitro* adventitious shoot production in Paulownia. Plant Cell Rep..

[17] Shurganov B.V., Mishutkina Y.V., Neskorodov Y.B. (2015). Development of an effective regeneration system for *Paulownia Shan Tong* (*P. fortunei* × *P. tomentosa*). Bull. RUDN Univ. Agron. Livest. Series. Sect. Biotechnol..

[18] Vasil I.K. (1987). Developing cell and tissue culture systems for the improvement of cereal and grass crops. J. Plant Physiol..

[19] Williams E.G., Maheswaran G. (1986). Somatic embryogenesis: Factors influencing coordinated behavior of cells as an embryogenic group. Ann. Bot..

[20] Jiménez V.M. (2001). Regulation of *in vitro* somatic embryogenesis with emphasis on to the role of endogenous hormones. Rev. Bras. Fisiol. Veg..

[21] Bishimbayeva N., Yertayeva B., Amirova A., Rakhimbayev I. (2017). Morphogenesis in Tissue Culture of Local Kazakh Cotton Varieties. OnLine J. Biol. Sci..

[22] Ipekci Z., Altinkut A., Kazan K., Bajrovic K., Gozukirmizi N. (2001). High Frequency Plant Regeneration from Nodal Explants of *Paulownia Elongate*. Plant Biol..

[23] Ahmed G., Roy P.K., Mamun A.N.K. (2001). High frequency shoot regeneration from nodal and shoot tip explants in *Holarrhena antidysenterica* L. Indian J. Exp. Biol..

[24] Murashige T., Skoog F. (1962). A Revised Medium for Rapid Growth and Bio Assays with Tobacco Tissue Cultures. Physiol. Plantarum..

[25] Chunchukov A., Yancheva S. (2015). Micropropagation of *Paulownia* species and hybrids. First National Conference of Biotechnology, Sofia. Annu. L’université Sofia “St. Kliment Ohridski” Fac. Biol..

[26] Roy P.K. (2015). *In vitro* plant regeneration of *Paulownia tomentosa* (Thunb.) Steud. from shoot tip and leaf segment. Bangladesh J. Bot..

[27] Shtereva L., Vassilevska-Ivanova R., Karceva T., Kraptchev B. (2014). Micropropagation of six *Paulownia* genotypes through tissue culture. J. Cent. Eur. Agric..

[28] Corredoira E., Ballester A., Vieitez A. (2008). Thidiazuron-Induced High-Frequency Plant Regeneration from Leaf Explants of *Paulownia tomentosa* Mature Trees. Plant Cell Tissue Organ Cult..

[29] Litwinczuk W., Bochnia E. (2012). Development of royal paulownia (*Paulownia tomentosa* Steud.) *in vitro* shoot cultures under the influence of different saccharides. Acta Sci. Pol. Hortorum Cultus..

[30] Taha L.S., Soad Ibrahim M.M., Farahat M.M. (2008). A micropropagation of *Paulownia* kowa-kami through *in vitro* culture technique. Austral. J. Basic Appl. Sci..

[31] Ipekci Z., Gozukirmizi N. (2003). Direct Somatic Embryo-genesis and Synthetic Seed Production from *Paulownia elongate*. Cell Biol. Morphog..

[32] Ipekci Z., Gozukirmizi N. (2005). Indirect Somatic Embryogenesis and Plant Regeneration from Leaf and Inter- node Explants of *Paulownia elongate*. Plant Cell Tissue Organ Cult..

[33] Pozoga M., Olewnicki D., Jablonska L. (2019). *In vitro* propagation protocols and variable cost comparison in commercial production for *Paulownia tomentosa* × *Paulownia fortune* hybrid as a renewable energy source. Appl. Sci..

[34] Rahman M.A., Rahman F., Rahmatullah M. (2013). *In vitro* regeneration of *Paulownia tomentosa* Steud. plants through the induction of adventitious shoots in explants derived from selected mature trees, by studying the effect of different plant growth regulators. Am. -Eurasian J. Sustain. Agric..

[35] Hussien E.T. (2020). Production of transgenic *Paulownia tomentosa* (Thunb.) steud using chitosan nanoparticles to express antimicrobial genes resistant to bacterial infection. Mol. Biol. Res. Commun..

[36] Kalinin F.L., Sarnatskaya V.V., Polishchuk V.E. (1980). Methods of Tissue Culture in Plant Physiology and Biochemistry.

[37] Dehestani-Ardakani M., Hejazi M., Aliabad K.K. (2020). Indirect somatic embryogenesis of purple conefower (*Echinacea purpurea* (L.) Moench): A medicinal-ornamental plant: Evaluation of antioxidant enzymes activity and histological study. Mol. Biol. Rep..

[38] Ahmadpour R., Zare N., Asghari-Zakarta R., Sheikhzadeh P. (2016). Efficient *In Vitro* Somatic Embryogenesis and Plant Regeneration from Mature and Immature Embryos of Wheat (*Triticum aestivum* L.). Braz. Arch. Biol. Technol..

[39] Khajuria A.K., Hano C., Bisht N.S. (2021). Somatic Embryogenesis and Plant Regeneration in *Viola canescens* Wall. Ex. Roxb.: An Endangered Himalayan Herb. Plants.

